# Growth conditions trigger genotype-specific metabolic responses that affect the nutritional quality of kale cultivars

**DOI:** 10.1093/jxb/erae169

**Published:** 2024-04-17

**Authors:** Hirofumi Ishihara, Sara Alegre, Jesús Pascual, Andrea Trotta, Wei Yang, Baoru Yang, Fatemeh Seyednasrollah, Meike Burow, Saijaliisa Kangasjärvi

**Affiliations:** Faculty of Biological and Environmental Sciences, Organismal and Evolutionary Biology Research Programme, 00014 University of Helsinki, Helsinki, Finland; Molecular Plant Biology, Department of Life Technologies, University of Turku, 20014, Turku, Finland; Molecular Plant Biology, Department of Life Technologies, University of Turku, 20014, Turku, Finland; Molecular Plant Biology, Department of Life Technologies, University of Turku, 20014, Turku, Finland; Institute of Bioscience and BioResources (IBBR), National Research Council of Italy (CNR), via Madonna del Piano, 10, 50019 Sesto Fiorentino (FI), Italy; Food Sciences, Department of Life Technologies, University of Turku, 20014 Turku, Finland; Food Sciences, Department of Life Technologies, University of Turku, 20014 Turku, Finland; Institute of Biotechnology, HILIFE – Helsinki Institute of Life Science, University of Helsinki, Helsinki, Finland; DynaMo Center, Department of Plant and Environmental Sciences, Faculty of Science, University of Copenhagen, Thorvaldsensvej 40, 1871 Frederiksberg C, Denmark; Faculty of Biological and Environmental Sciences, Organismal and Evolutionary Biology Research Programme, 00014 University of Helsinki, Helsinki, Finland; Faculty of Agriculture and Forestry, Department of Agricultural Sciences, 00014 University of Helsinki, Helsinki, Finland; Viikki Plant Science Centre, 00014 University of Helsinki, Helsinki, Finland; University of Birmingham, UK

**Keywords:** Anthocyanin, *Brassica oleracea*, glucosinolate, indoor cultivation, kale, metabolite profiling, nutritional quality, transcriptome

## Abstract

Kales (*Brassica oleracea* convar. *acephala*) are fast-growing, nutritious leafy vegetables ideal for year-round indoor farming. However, selection of the best cultivars for growth under artificial lighting necessitates a deeper understanding of leaf metabolism in different kale types. Here we examined a curly-leaved cultivar, Half Tall, and a lacinato-type cultivar, Black Magic, under moderate light (130 µmol photons m^−2^ s^−1^/22 °C) and high light (800 µmol photons m^−2^ s^−1^/26 °C) conditions. These conditions induced genotype-dependent differences in nutritionally important metabolites, especially anthocyanins and glucosinolates (GSLs), in kale cultivars. In the pale green Half Tall, growth under high light conditions did not induce changes in either pigmentation or total GSL content. In contrast, the purple pigmentation of Black Magic intensified due to increased anthocyanin accumulation. Black Magic showed reduced contents of indole GSLs and increased contents of aliphatic GSLs under high light conditions, with notable cultivar-specific adjustments in individual GSL species. Correlation analysis of metabolite profiles suggested cultivar-specific metabolic interplay between serine biosynthesis and the production of indole GSLs. RNA sequencing identified candidate genes encoding metabolic enzymes and regulatory components behind anthocyanin and GSL biosynthesis. These findings improve our understanding of leaf metabolism and its effects on the nutritional quality of kale cultivars.

## Teaser

High light coupled with elevated temperature induces genotype-specific metabolic responses in kale, suggesting that tailoring lighting conditions to cultivars can enhance crop nutritional quality in indoor horticulture.

## Introduction

*Brassica oleracea* is an important vegetable species that originated from Mediterranean and Atlantic coastal areas of Europe (Smyth, 1995). It has been dined on since at least 2000 bc, and its selective cultivation has given rise to different *B. oleracea* sub-species, including cabbage (var *capitata*), kohlrabi (var *gongylodes*), Brussel sprouts (var *gemmifera*), broccoli (var *italica*), and kale (var *acephala*; Smyth, 1995). Today, kales are among the most produced leafy vegetables worldwide (FAO, 2021). Studies on kale cultivars have reported diverse coloration, size, and texture of leaves and identified metabolic differences in the contents of glucosinolates (GSLs) (Christensen *et al*., 2011; Hahn *et al*., 2016; Arias *et al*., 2021) and anthocyanins (Mageney *et al*., 2017). Owing to their high contents of minerals, vitamins, and specialized metabolites, kales represent an attractive option for year-round indoor farming. However, maximizing the benefits of indoor cultivation necessitates a deeper understanding of how growth conditions affect the productivity and nutritional value of kale cultivars.

Plants respond to light by transient photosynthetic adjustments and more durable metabolic and morphological responses (Aro *et al*., 1993; Tikkanen *et al.*, 2012; Pascual *et al*., 2017; Foyer, 2018; Thoma *et al.*, 2020). Under low light, relative chlorophyll content increases to enhance photosynthetic light harvesting, while contents of photoprotective β-carotene and xanthophylls decline (Anderson, 1986; Demmig-Adams *et al*., 1996). Hence, a decrease in the carotenoid/chlorophyll ratio is typically observed in shaded plants (Demmig-Adams *et al*., 1996). Under increasing light intensities, a typical response is accumulation of anthocyanins, which are water-soluble, antioxidative phenylpropanoid compounds that evolved to protect plants against UV-light (Castañeda-Ovando *et al.*, 2009). Anthocyanin pigments are typical in red, purple, and blue vegetables and fruits and they have been associated with sensory attributes of astringency and bitterness (Paissoni *et al.*, 2018). In human diet, consumption of anthocyanin-rich food maintains antioxidant capacity and eases oxidative stress in chronic diseases (Hurst *et al.*, 2020).

**Fig. 1.**
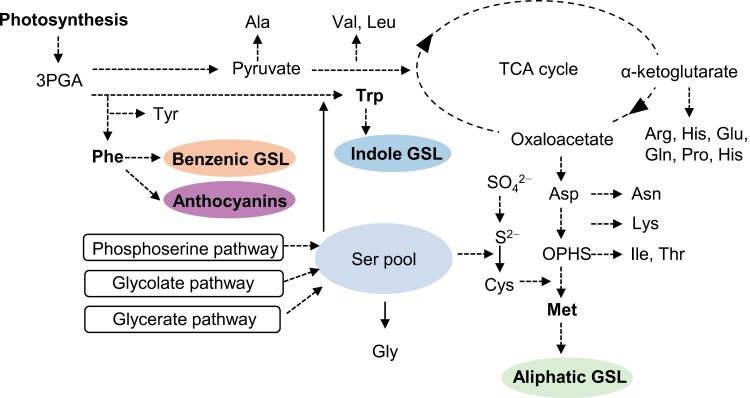
Schematic representation of amino acid biosynthesis and its metabolic links with glucosinolates and anthocyanins. Ser contributes to the biosynthesis of Met and Trp through three distinct biosynthetic pathways (Ros *et al.*, 2014). Met and Trp serve as precursors for aliphatic and indole glucosinolates (GSLs), respectively. The dotted lines indicate multiple reactions. OPHS, *O*-phosphohomoserine.

Anthocyanins are synthesized from Phe, which serves as a precursor for the phenylpropanoid pathway, which is conserved among plant plants (Liu *et al*., 2018; Fig. 1). Additional side pathways that emerged from various intermediates of the core pathway introduced diverse phenylpropanoid profiles in land plants (Tohge *et al*., 2016), with more than 600 anthocyanin derivatives identified to date (Smeriglio *et al*., 2016). Stressful combinations of light and heat can induce the accumulation of anthocyanins (Chalker-Scott, 1999; Zeng *et al*., 2010), but exposure to heat stress alone led to reduced anthocyanin biosynthesis in Arabidopsis (Kim *et al*., 2017). Hence, leaf pigmentation is intricately regulated in response to prevailing environmental cues.

While phenylpropanoids are common to all land plants, GSLs are S- and N-containing specialized metabolites characteristic of the family of *Brassicaceae* (Clarke, 2010). Studies on the *Brassicaceae* family have unveiled the significant commercial and ecological implications of GSLs in human and animal nutrition, as well as their role in plant–environment interactions, including interactions with pathogens and herbivores (Wittstock and Burow, 2010; Traka, 2016; Francisco *et al*., 2017). The basic GSL skeleton consists of a β-thioglucose residue, an *N*-hydroxy monosulfate moiety and a variable amino acid-derived side chain (Halkier and Du, 1997; Kliebenstein *et al*., 2001b). About 130 GSLs have so far been identified and characterized in plants (Blažević *et al*., 2020). Studies on Arabidopsis have elucidated the biosynthesis, modification, degradation, and transport of certain GSLs (Halkier and Gershenzon, 2006; Sønderby *et al*., 2010; Jensen *et al.*, 2014) and uncovered mechanisms behind the transcriptional and post-translational regulation of these processes (Celenza *et al.*, 2005; Gigolashvili *et al.*, 2007; Frerigmann *et al.*, 2016; Rahikainen *et al.*, 2017; Millard *et al*., 2019).

GSLs are divided into three different classes based on their amino acid precursors, called benzenic GSLs (mainly derived from Phe), aliphatic GSLs (mainly derived from Met), and indole GSLs (mainly synthesized from Trp) (Fig. 1) (Blažević *et al*., 2020; Windsor *et al*., 2005). The biosynthesis of Met and Trp consumes Ser as a metabolic precursor. In plants, Ser can derive from three different biosynthetic pathways: the glycolate pathway (photorespiration), the glycerate pathway (cytosolic glycolysis), and the phosphoserine pathway (Ros *et al.*, 2014). In Arabidopsis, maintenance of active indole GSL biosynthesis required the activity of the phosphoserine pathway for Ser biosynthesis (Zimmermann *et al*., 2021). It was therefore proposed that the phosphoserine pathway could supply Ser for the biosynthesis of Trp under conditions of high indole GSL biosynthesis demand (Fig. 1) (Zimmermann *et al*., 2021).

The structural diversity of GSLs stems from modifications that may occur in both the initial elongation of the side chain of some amino acid precursors and/or enzymatic modification of the GSL side chain (Sønderby *et al*., 2010; Jeschke and Burow, 2018). Upon cell disruption, the thioglucosidic bond is hydrolysed by thioglucosidases, and subsequent chemical and enzymatic reactions lead to the formation of bioactive compounds, including isothiocyanates, nitriles, oxazolidine-2-thiones, and thiocyanate (Wittstock and Burrow 2010; Blažević *et al*., 2020). Isothiocyanates are highly reactive organosulfur phytochemicals that have been widely studied in human nutrition (Lee *et al.*, 2020). Consumption of GSL-rich *Brassica* crops has been associated with a reduced risk of cancer and chronic inﬂammation diseases, with the beneficial health effects mostly linked to isothiocyanates, such as sulforaphane (Connolly *et al*, 2021). In contrast, oxazolidine-2-thiones and thiocyanate, derivatives of progoitrin and indole GSL, respectively, can impede thyroid function and elevate the risk of goiters (Felker *et al*., 2016). Since some of the GSL species and their degradation products are beneficial, while others have negative effects, the GSL composition of cruciferous vegetables is highly relevant for human nutrition.

Here we set out to assess how growth under different light and temperature conditions affects the nutritional value of curly leaved (cv. Half Tall) and lacinato (cv. Black Magic) type kales. We report that growth under moderate growth light (130 µmol photons m^−2^ s^−1^/22 °C) and high light (800 µmol photons m^−2^ s^−1^/26 °C) conditions induced genotype-specific changes in the profiles of GSL and anthocyanins. In addition, we pinpoint candidate genes and metabolic interactions that may affect the underlying biosynthetic processes in differentially light and temperature-acclimated kale cultivars. Our findings suggest that optimizing the growth environment in a cultivar-specific manner can significantly affect the quality of plant-based food.

## Materials and methods

### Plant material, growth light and temperature conditions

*Brassica oleracea* convar. *acephala*, cv. Half Tall and cv. Black Magic were cultivated in a growth room at 50% relative humidity and 12/12 h photoperiod under Osram PowerStar HQI-T 400/D metal-halide lamps (Osram Licht AG; Munich, Germany), as described by Piippo *et al.* (2006). Plants were first germinated in control light (CL) conditions (130 µmol photons m^−2^ s^−1^/22 °C) for 2 d, and thereafter either kept in control light conditions or transferred to high light (HL) conditions, which was accompanied by a temperature elevation to 26 °C (800 µmol m^−2^ s^−1^/26 °C). Note, because of the recorded 4 °C temperature increase, the term high light condition here refers to a combination of high light intensity and elevated temperature.

The experiments were carried out with 19-day-old plants that were randomized during growth. Leaf samples were harvested 4 h into the light period. The data were analysed with three to eight biological replicates per light condition, as indicated in the figure legends. Each biological replicate consisted of longitudinal halves of two independent leaves that were cut though the midrib. This sampling procedure allowed dividing the same leaf material for metabolomic and RNA-seq analysis.

### Spectrophotometric measurement of total leaf pigments

Spectrophotometric quantification of kale leaf pigments was performed as described in Sims and Gamon (2002). Chlorophyll, carotenoids, and anthocyanins were extracted and measured as described in Sims and Gamon (2002).

### Mass spectrometric analysis of anthocyanins

Anthocyanins were extracted and quantified using an UPLC-ESI-MS/MS apparatus (Bruker Corporation, Billerica, MA, USA), as specified in Yang *et al.* (2018). An external standard of cyanidin 3-*O*-glucoside was used for quantitative analysis, and all the anthocyanins were quantified as equivalents of cyanidin 3-*O*-glucoside, using the calibration curve constructed with this reference compound. The total content of anthocyanins was calculated as the sum of the peaks in the chromatogram. The detected anthocyanins were tentatively identified based on mass spectra, UV spectra, and comparison with previously published literature (Table 1; Supplementary Fig. S1).

**Table 1.** Anthocyanins identified in kale cultivar Black Magic leaves

Number	Retention time (min)	*m*/*z*	Product [MS^2^]	Tentative ID
1	9.18	979	817, 449, 287	Cyanidin-3-sinapoyl-diglucoside-5-glucoside
2	9.96	949	449, 287	Cyanidin-3-feruloyl-diglucoside-5-glucoside
3	14.72	1288	1126, 617, 449, 287	Unknown
4	14.95	1141	979, 653, 449, 287	Cyanidin-3-sinapoyl-caffeoyl-diglucoside-5-glucoside I
5	15.88	1141	979, 653, 449, 287	Cyanidin-3-sinapoyl-caffeoyl-diglucoside-5-glucoside II
6	16.16	949	449, 287	Cyanidin-3-caffeoyl-feruloyl-diglucoside
7	16.82	1125	963, 449, 287	Cyanidin-3-sinapoyl-p-coumaroyl-diglucoside-5-glucoside
8	16.93	1111	930, 287	Unknown
9	17.09	1155	963, 449, 287	Cyanidin-3-feruloyl-sinapoyl-diglucoside-5-glucoside
10	17.35	1185	1023, 449, 287	Cyanidin-3-disinapoyl-diglucoside-5-glucoside

The numbers refer to the peaks obtained in the chromatography analysis presented in Supplementary Fig. S1. *m*/*z*, mass-to-charge ratio, [M+H]^+^.

### RNA extraction and RNA sequencing library construction

Total RNA was isolated from homogenized fresh-frozen plant leaf materials from four biological replicates per treatment per genotype, using the innuPREP Plant RNA kit (Analytik Jena AG, Jena, Germany). The purity of the RNA was assessed, using a NanoPhotometer (Implen Inc., Westlake Village, CA, USA), and the RNA integrity and quantity were thereafter assessed using the RNA Nano 6000 assay kit of the Bioanalyzer 2100 system as described in the manual (Agilent Technologies Inc., Santa Clara, CA, USA). The RNA-seq libraries from the kale cultivars were constructed by Novogene (Cambridge, UK). Briefly, a total amount of 1 µg RNA per sample was used to construct RNA-seq libraries using NEBNext Ultra^TM^ RNA Library Prep Kit for Illumina (New England Biolabs, Ipswich, MA, USA). The total RNA was fragmented at 65 °C for 5 min, followed by poly(A) enrichment with oligo (dT)_25_ magnetic beads (New England Biolabs). Double stranded cDNA was synthesized using random hexamer primer and M-MuLV Reverse Transcriptase (New England Biolabs), adenylated, and ligated with NEBNext adaptor. cDNA fragments of 150~200 bp in length were purified using AMPure X Pbeads (Beckman Coulter, Brea, CA, USA). The hairpin loop of NEBNext adaptor was opened using USER enzyme (New England Biolabs) prior to PCR with universal PCR primers and index primers. The Agilent Bioanalyzer 2100 system was the used to assess the quality of the constructed RNA-seq library. Clustering of the index-coded samples was performed on a cBot Cluster Generation System using PE Cluster Kit cBot-HS (Illumina) according to the manufacturer’s instructions.

### RNA-seq data processing and reference-based differential gene-expression analysis

The libraries were sequenced on the Illumina HiSeq 2500 platform by Novogene (Cambridge, UK). The quality of the raw paired-end sequencing reads was assessed using the FastQC tool (Andrews, 2010). When necessary, the raw reads were pre-processed, using the Trimmomatic tool (ver. 0.39) to ensure removal of the Illumina adapters and trimming of the low-quality bases (Bolger *et al.*, 2014). The clean reads were then mapped against 29980 reference coding sequences (CDS) from *Brassica oleracea* var. *capitata* (cabbage) from Cai *et al.* (2020), using the STAR aligner (ver. 2.7.8a; Dobin *et al.*, 2013; Cai *et al.*, 2020). Simultaneously, gene-expression abundances (counts per million; CPM) were estimated, using the -quantMode GeneCounts option (Dobin *et al*., 2013).

Statistical analysis of the differential gene expression was conducted utilizing the Limma R-Bioconductor package (ver. 3.48.3; Ritchie *et al*., 2015). Prior to differential expression testing, genes expressed at low level (CPM value <0.5) were excluded from the analysis, while the expression levels of the remaining genes were normalized for sequencing depth and RNA composition, using the trimmed mean of M-values method implemented in the edgeR R-Bioconductor package (ver. 3.34.1; Robinson and Oshlack, 2010; Robinson *et al*., 2010). The false discovery rate (FDR) method provided by Limma was used to adjust for multiple testing issues. An FDR <0.05 and an absolute value of log_2_ fold-change (fc) >1 were used as cut-off criteria to generate the final list of statistically significant differentially expressed genes (DEGs). The similarity of the gene-expression profiles across sample groups was assessed, using a multidimensional scaling (MDS) method plot implemented in the Limma package. Mercator4 was used to assign functional annotations to the cabbage CDS sequences for MapMan bin enrichment analysis (Schwacke *et al*., 2019; Cai *et al.* 2020). MapMan bin enrichment was analysed, using web-based software in the PlaBi database (https://plabipd.de/portal/bin-enrichment).

### Glucosinolate analysis

To analyse the GSL composition of the kale leaves, the leaf samples were ground in liquid nitrogen. Homogenized plant leaf material was used to extract GSL as delsulfo-GSL (dsGLS), as previously described (Crocoll *et al.*, 2016). The dsGSLs were analysed using ultra high performance liquid chromatography (UHPLC) coupled to an EVOQ Elite triple quad (TQ) mass spectrometer with an electrospray ionization (ESI) source (UHPLC-ESI-TQ-MS/MS). The individual GLSs were quantified based on response factors relative to the internal standard *p*-OH-benzyl-GSL calculated from standard curves in control extracts, as described (Crocoll *et al.*, 2016).

### Amino acid analysis

Amino acids were extracted and measured by UHPLC-ESI-TQ-MS/MS, as previously described (Petersen *et al*., 2019). Response factors for quantification of amino acids had been calculated previously based on dilution series of the respective analytes (Petersen *et al*., 2019).

### Statistical analyses of metabolite data

All the statistical analyses were performed in the R environment ver. 3.5.1 (R Core Team, 2018). Numerical data obtained from analysis of amino acids, GSLs, and total pigments were subjected to statistical analysis using two-way ANOVA with statistical significance at the level of *P*<0.05, followed by post-hoc pairwise comparisons. For post-hoc pairwise comparisons, *P*-values were corrected for multiple comparisons using Bonferroni adjustment.

## Results

### Cultivar-specific changes in leaf pigmentation in response to light and temperature conditions

**Fig. 2.**
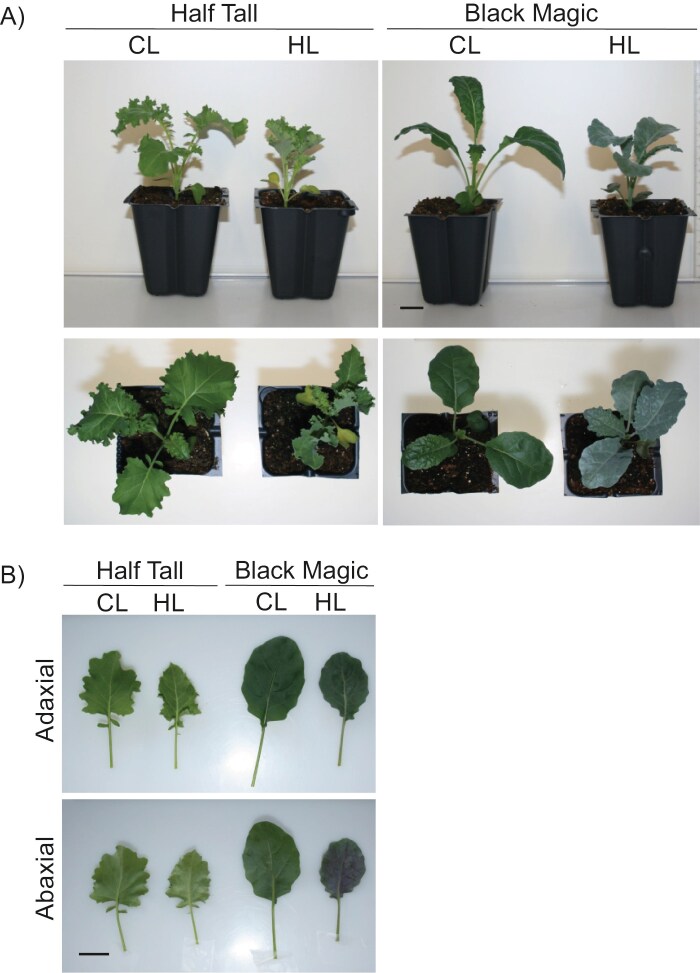
Visual characteristics of kale (*Brassica oleracea* convar. *acephala*) cultivars Half Tall and Black Magic. (A) Morphological characteristics of kale cultivars Half Tall and Black Magic after 3 weeks’ growth under 130 µmol photons m^−2^ s^−1^ at 22 °C [control light (CL) conditions] or under 800 µmol photons m^−2^ s^−1^ at 26 °C [high light (HL) conditions]. The scale bars correspond to 2 cm. (B) Photographs depicting adjustments in leaf morphology and pigmentation as visualized from adaxial and abaxial surfaces of kale leaves. The scale bars correspond to 2 cm.

**Fig. 3.**
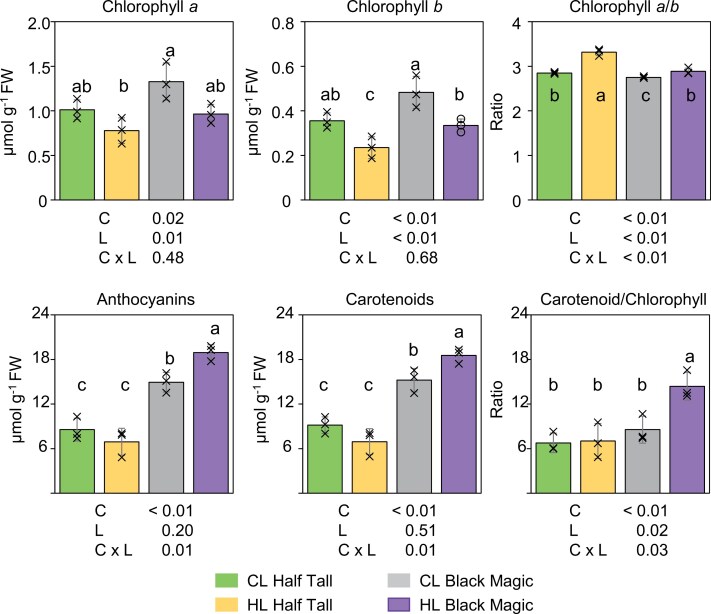
Effects of light and temperature conditions on leaf pigment composition in kale cultivars Black Magic and Half Tall. Spectrophotometric quantification of chlorophyll *a*, *b*, total carotenoid, and anthocyanin contents in kale cultivars Half Tall and Black Magic grown under control light conditions [CL, 130 µmol photons m^−2^ s^−1^ at 22 °C] or under high light conditions [HL, 800 µmol photons m^−2^ s^−1^ at 26 °C]. The *P*-values from the two-way ANOVA (~cultivar (C) + light condition (L) + C×L) are indicated below the graphs. For post-hoc pairwise comparisons, *P*-values were corrected for multiple comparisons using Bonferroni adjustment. Different letters indicate statistically significant differences (significance threshold, *P*=0.05). The error bars indicate the standard deviation (*n*=3). The data are presented in Supplementary Dataset S1.

The *Brassica oleracea* convar*. acephala* cultivars Half Tall and Black Magic were grown under control light conditions (130 µmol photons m^−2^ s^−1^ and 22 °C) or high light conditions (800 µmol photons m^−2^ s^−1^ and 26 °C). After growing the kales for 19 d under high light conditions, the purple pigmentation of Black Magic intensified in contrast to the pale green colour of the Half Tall leaves (Fig. 2). Spectrophotometric analysis of Half Tall and Black Magic leaf extracts displayed typical high-light-induced responses with decreasing chlorophyll content and increasing chlorophyll *a*/*b* ratio in both cultivars (Fig. 3; Supplementary Dataset S1). However, elevated contents of anthocyanins and carotenoids, and increased carotenoid/chlorophyll ratios were only found in high-light-grown Black Magic leaves, but not in Half Tall. These findings indicated that growth under different light and temperature conditions induced differential effects amongst the kale cultivars (Fig. 3).

For a more detailed assessment of the anthocyanin contents, the kale leaf extracts were analysed using an UPLC-ESI-MS/MS (Table 1; Supplementary Fig. S1). In Half Tall, only trace amounts of anthocyanins were detected, and individual compounds could therefore not be reliably identified. In contrast, 10 different cyanidin-derived compounds were quantitatively identified in Black Magic (Table 1). The anthocyanins were found in different acylated forms, with sinapic acid, ferulic acid, caffeic acid, and *p*-coumaric acid as the predominant acyl donors (Table 1). Compounds 9 and 10, tentatively identified as cyanidin-3-sinapoyl-feruloyl-diglucoside-5-glucoside and cyanidin-3-disinapoyl-diglucoside-5-glucoside, respectively, were the most abundant anthocyanins detected in Black Magic (Table 1; Supplementary Dataset S2). UPLC-ESI-MS/MS-based quantification of anthocyanins subjected to one-way ANOVA revealed that the levels of different anthocyanin derivatives in Black Magic significantly increased upon acclimation to high light conditions, as compared with Black Magic grown under control light conditions (adjusted *P*=0.014).

### Cultivar-specific accumulation of glucosinolates under different light conditions

**Fig. 4.**
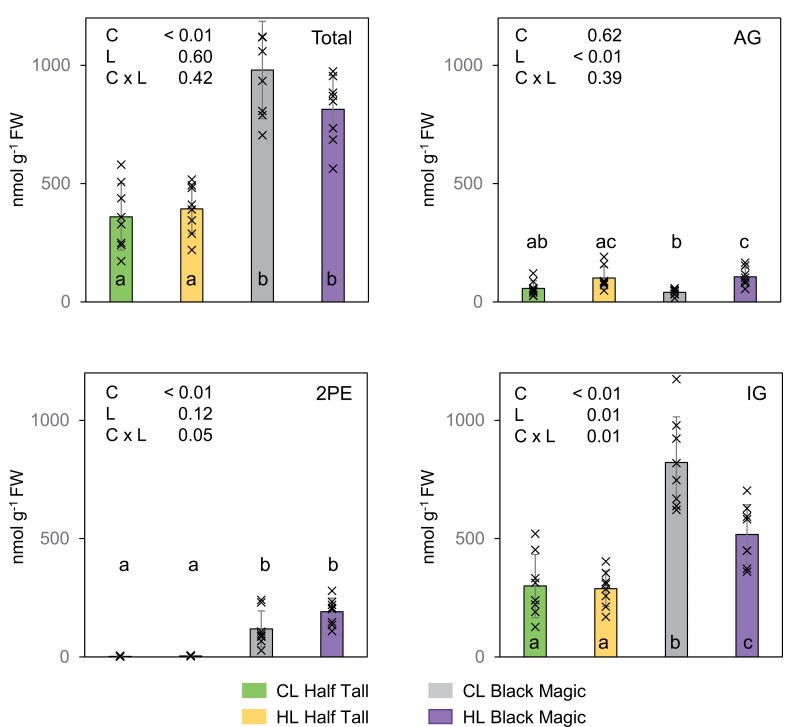
Glucosinolate contents in the leaves of differentially light-acclimated kale cultivars Half Tall and Black Magic. Kale cultivars Half Tall and Black Magic were grown under 130 µmol photons m^−2^ s^−1^ at 22 °C (CL) or 800 µmol photons m^−2^ s^−1^ at 26 °C (HL). Data are shown as means ±SD (*n*=8). The *P*-values from two-way ANOVA (~cultivar (C) + light condition (L) + C×L) are indicated in the upper left corner. For post-hoc pairwise comparisons, *P*-values were corrected for multiple comparisons using Bonferroni adjustment (significance threshold, *P*=0.05). Different letters indicate significant differences between sample groups. 2PE, 2-phenylethylglucosinolate; AG, aliphatic glucosinolates; IG, indole glucosinolates; Total, total glucosinolate levels.

Next, we examined how acclimation to different growth conditions affected the contents of glucosinolates, a group of specialized metabolites particularly abundant in *Brassica* crops (Hahn *et al.*, 2016). The UPLC-ESI-MS/MS analysis identified eight aliphatic GSLs, three indole GSLs, and one benzenic GSL (Table 2). The most abundant GSLs were indole GSLs representing 63% and 84% of the total GSL contents in Half Tall and Black Magic, respectively (Fig. 4). Neither Half Tall nor Black Magic displayed light condition-dependent changes in the total contents of GSLs (Fig. 4). However, the growth light and temperature conditions induced significant changes in the composition of GSLs (Table 2).

**Table 2.** Quantification of glucosinolates in differentially light-acclimated in kale leaves

	Glucosinolate content (nmol g^−1^ FW)						
	cv. Half Tall		cv. Black Magic		Two-way ANOVA *P*-value		
	CL	HL	CL	HL	C	L	C×L
3MTP	1.53 ± 1.02	5.71 ± 3.18	n.d.	n.d.	n.a.	0.01	n.a.
3MSP	9.09 ± 4.91	16.5 ± 10.6	3.19 ± 3.51	1.76 ± 0.80	<0.01	0.12	0.15
2PROP	37.43 ± 28.9	49.2 ± 36.4	0.00 ± 0.00	0.00 ± 0.00	n.a.	0.66	n.a.
4MTB	n.d.	1.13 ± 0.20	0.78 ± 0.18	1.73 ± 0.57	<0.01	<0.01	0.01
4MSB	4.61 ± 3.14	20.3 ± 5.32	38.1 ± 12.5	101.3 ± 37.7	<0.01	<0.01	<0.01
3BUT	1.22 ± 0.71	3.08 ± 1.14	n.d.	n.d.	n.a.	<0.01	n.a.
2*R*-2OH-3BUT	2.73 ± 2.2	4.79 ± 3.16	n.d.	n.d.	n.a.	0.01	n.a.
5MSP	1.04 ± 0.37	1.48 ± 0.36	1.64 ± 0.55	1.72 ± 0.54	0.25	0.71	0.9
Total AG	57 ± 32.3	100.9 ± 47.9	40.8 ± 12.87	105.7 ± 38	0.01	<0.01	0.06
I3M	274 ± 120.8	256.9 ± 73.9	778.8 ± 195.8	446.8 ± 126.5	<0.01	0.01	0.01
4MOI3M	2.59 ± 0.90	1.41 ± 0.44	2.79 ± 0.97	1.41 ± 0.35	0.86	<0.01	0.86
1MOI3M	23.1 ± 18.3	30.3 ± 7	39.5 ± 9.9	68.9 ± 27.2	<0.01	0.03	0.07
Total IG	299.7 ± 133.9	288.6 ± 75.5	821.2 ± 193.5	517.1 ± 126.3	<0.01	0.01	0.01
2PE	2.72 ± 1.76	3.86 ± 1.65	118.2 ± 76.5	191.5 ± 56.7	<0.01	0.21	0.01
Total GSLs	359.4 ± 140.8	393.4 ± 104.8	980.2 ± 206	814.3 ± 141.7	<0.01	0.6	0.42

CL: control light (130 µmol photons m^−2^ s^−1^ at 22 °C), HL: high light condition (800 µmol photons m^−2^ s^−1^ at 26 °C). The data are means ±SD, *n*=8. n.a., not analysed; n.d., values that were below the lower level of quantification. Two-way ANOVA was used to test for potential effects of cultivar (C), light condition (L), and C×L interaction (significance threshold, *P*=0.05). The source data are presented in Supplementary Dataset S3. 1MOI3M, 1-methoxyindol-3-ylmethyl GSL; 2PE, gluconasturtiin, 2-phenylethylglucosinolate GSL; 2PROP, sinigrin, 2-propenyl GSL; 2*R*-2OH-3BUT, progoitrin, 2(*R*)-2-hydroxy-3-butenyl GSL; 3BUT, gluconapin, 3-butenyl GSL; 3MSP, glucoiberin, 3-methylsulfinylpropyl GSL; 3MTP, glucoiberverin, 3-methylthiopropyl GSL; 4MOI3M, 4-methoxyglucobrassicin, 4-methoxyindol-3-ylmethyl GSL; 4MSB, glucoraphanin, 4-methylsulfinylbutyl GSL; 4MTB, glucoerucin, 4-methylthiolbutyl GSL; 5MSP, glucoalyssin, 5-methylsulfinylpentyl GSL; AG, aliphatic glucosinolates; AOP2, alkenyl hydroxalkyl producing 2; I3M, glucobrassicin, indol-3-ylmethyl GSL; IG, indole glucosinolates; GSL, glucosinolate.

In comparison with Half Tall, Black Magic contained twice the amount of GSLs, which was attributed to higher contents of indole GSLs, and over 40 times higher gluconasturtiin (2PE; 2-phenylethyl GSL) (Fig. 4; Table 2). The contents of total aliphatic GSLs were similar between the cultivars (Fig. 4; Table 2). Upon acclimation to high light and elevated temperature, the aliphatic GSL content increased significantly in Black Magic (Fig. 4; Table 2). In Black Magic, the increase in apliphatic GSLs was accompanied by decreased content of indole GSLs (*P*=0.014; Fig. 4). The lower content of indole GSLs in Black Magic under high light conditions was due to a decrease in glucobrassicin (I3M, indolyl-3-ylmethyl GSL), which constituted about 90% of total indole GSLs (Table 2; Supplementary Fig. S2). No significant change of gluconasturtiin (2PE, 2-phenylethylglucosinolate GSL) was found in either of the cultivars in high light conditions (Fig. 4).

The biosynthesis of aliphatic GSLs comprises three main stages: elongation of the amino acid chain (for some amino acid precursors), formation of the core GSL structure, and modification of the side chain. As expected for *Brassica* species (Verkerk *et al*., 2009), aliphatic GSLs with C3–C5 (referring to the number of carbons in their aliphatic side chains) were detected (Table 2; Supplementary Fig. S3). Quantitative analysis revealed genotype-dependent differences in aliphatic GSL profiles between the two cultivars. Half Tall displayed high contents of the C3 aliphatic GSLs glucoiberverin (3MTP, 3-methylthiopropyl GSL), glucoiberin (3MSP; 3-methylsulfinylpropyl GSL) and sinigrin (2PROP, 2-propenyl GSL). In contrast, Black Magic was rich in glucoraphanin (4MSB, 4-methylsulfinylbutyl GSL), while 2PROP was not detected in either of the lighting conditions (Table 2; Supplementary Fig. S3). Furthermore, gluconapin (3BUT, 3-butenyl GSL) and progoitrin (2*R*-2OH-3BUT, 2(*R*)-2-hydroxy-3-butenyl GSL) were only detected in Half Tall (Table 2; Supplementary Fig. S3).

### Correlation between amino acid and glucosinolate abundance

**Fig. 5.**
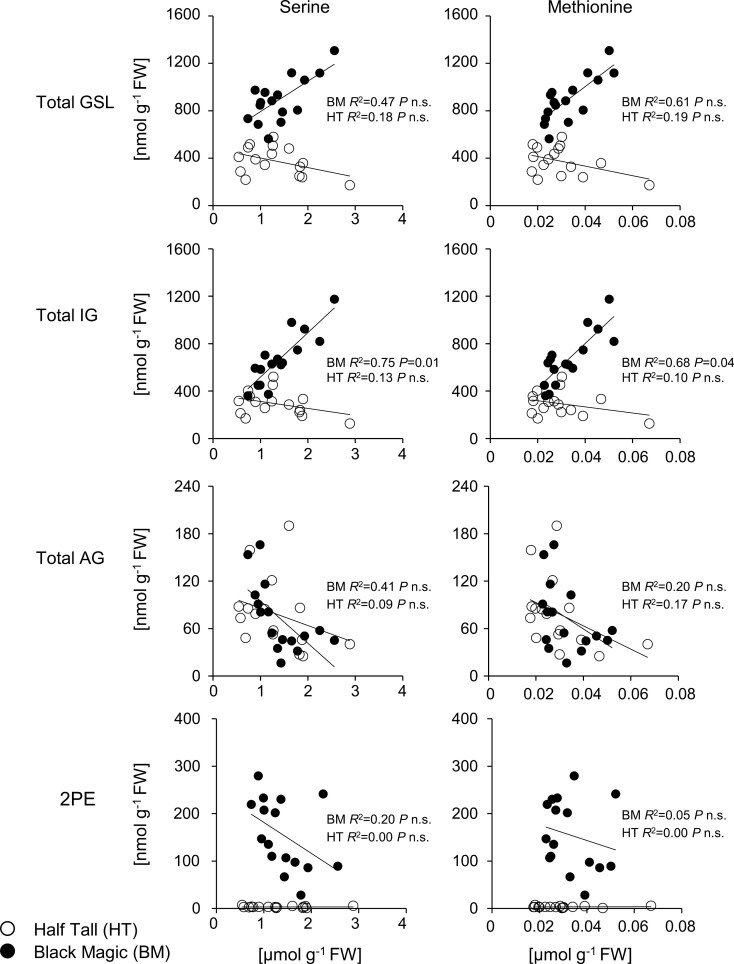
Association between amino acids and glucosinolates in the leaves of kale cultivars Half Tall and Black Magic. Summary of the association between the amino acids (Ser or Met) and total content of indole glucosinolate (IG), aliphatic glucosinolate (AG), benzenic glucosinolate (2PE), and total glucosinolats (GSL=IG+AG+2PE). The *P*-values indicate a statistically significant correlation between Met/Ser with GSL levels (*P*<0.05). The open and closed symbols represent single biological replicates of Half Tall and Black Magic, respectively. The original data are presented in Supplementary Dataset S3. The *P*-values were calculated from Pearson’s *R* and adjusted, using Bonferroni correction. The original data are presented in Supplementary Dataset S4.

The biosynthesis of GSLs is tightly linked with amino acid metabolism (Fig. 1). Within the metabolic networks, Ser is indirectly connected with indole GSL biosynthesis, as it serves as a metabolic precursor for Met and Trp (Benstein *et al.*, 2013; Zimmermann *et al*., 2021). This metabolic association prompted us to examine whether the availability of amino acid precursors correlated with GSL contents in kale leaves. Quantification of amino acids revealed that total amino acid contents of Black Magic and Half Tall were similar in control light conditions and reduced to less than half in high light with elevated temperature in both cultivars (Table 3). Both kales responded to the high light conditions with diminished contents of several amino acids, especially Asp, Gln, Glu, and Ser, which together comprised more than 80% of the total amino acids quantified in the samples (Table 3; Supplementary Dataset S3). In contrast, aromatic amino acids, including Trp and Phe, which serve as precursors for the biosynthesis of GSLs and anthocyanins, showed no changes in response to growth under high light and elevated temperature (Table 3). Two-way ANOVA revealed no cultivar effects on measured amino acid levels, except for Pro (Table 3; cultivar *P*<0.01). Pearson’s correlation coefficient analysis, using the data from both lighting conditions, displayed no significant correlation between amino acids and GLSs in Half Tall (Fig. 5; Supplementary Dataset S4). However, in Black Magic the contents of Met and Ser were significantly and positively correlated with the total indole GSL content (Bonferroni-corrected *P*-values; Met, *P*=0.04; Ser, *P*=0.01; Fig. 5).

**Table 3.** Quantification of amino acids in differentially light-acclimated kale leaves

	Amino acid content (µmol g^−1^ FW)						
	cv. Half Tall		cv. Black Magic		Two-way ANOVA adjusted *P*-value		
	CL	HL	CL	HL	C	L	C×L
Ala	0.42 ± 0.16	0.28 ± 0.10	0.34 ± 0.09	0.24 ± 0.06	0.19	0.01	0.78
Arg	0.03 ± 0.01	0.02 ± 0.00	0.03 ± 0.03	0.01 ± 0.00	0.54	0.02	0.32
Asn	0.22 ± 0.12	0.04 ± 0.01	0.11 ± 0.03	0.04 ± 0.01	0.03	<0.01	0.03
Asp	1.4 ± 0.55	0.54 ± 0.21	1.50 ± 0.45	0.57 ± 0.14	0.64	<0.01	0.78
Gln	6.05 ± 2.54	0.77 ± 0.46	4.69 ± 2.02	0.92 ± 0.25	0.46	<0.01	0.32
Glu	4.44 ± 1.63	2.72 ± 0.72	3.88 ± 0.85	2.59 ± 0.41	0.42	<0.01	0.64
His	0.08 ± 0.03	0.05 ± 0.01	0.06 ± 0.02	0.03 ± 0.01	0.06	<0.01	0.93
Ile	0.07 ± 0.02	0.04 ± 0.01	0.07 ± 0.03	0.04 ± 0.01	0.72	<0.01	0.75
Leu	0.02 ± 0.00	0.04 ± 0.02	0.01 ± 0.01	0.02 ± 0.01	0.10	0.03	0.31
Lys	0.03 ± 0.01	0.02 ± 0.01	0.02 ± 0.01	0.02 ± 0.01	0.32	0.01	0.60
Met	0.04 ± 0.01	0.02 ± 0.00	0.04 ± 0.01	0.03 ± 0.00	0.29	<0.01	0.45
Phe	0.02 ± 0.01	0.03 ± 0.01	0.02 ± 0.01	0.02 ± 0.01	0.08	0.63	0.53
Pro	0.27 ± 0.05	0.32 ± 0.06	0.19 ± 0.05	0.24 ± 0.04	0.00	0.04	0.68
Ser	1.76 ± 0.54	0.86 ± 0.35	1.80 ± 0.42	1.01 ± 0.16	0.48	<0.01	0.77
Thr	0.52 ± 0.21	0.19 ± 0.05	0.46 ± 0.14	0.20 ± 0.04	0.75	<0.01	0.58
Trp	0.00 ± 0.00	0.01 ± 0.00	0.00 ± 0.00	0.00 ± 0.00	0.29	0.18	0.33
Tyr	0.01 ± 0.00	0.02 ± 0.01	0.01 ± 0.00	0.02 ± 0.01	0.54	0.02	0.74
Val	0.13 ± 0.02	0.12 ± 0.03	0.13 ± 0.05	0.13 ± 0.03	0.73	0.78	0.41
Total AA	15.51 ± 5.67	6.06 ± 1.88	13.38 ± 3.99	6.14 ± 1.01	0.55	<0.01	0.51

CL: control light (130 µmol photons m^−2^ s^−1^ at 22 °C); HL: high light condition (800 µmol photons m^−2^ s^−1^ at 26 °C). The data are means ±SD, *n*=8. Two-way ANOVA was used to test for potential effects of cultivar (C), light condition (L), and C×L interaction (significance threshold, *P*=0.05). The source data are presented in Supplementary Dataset S3.

### RNA-seq transcript profiling of kale cultivars

**Fig. 6.**
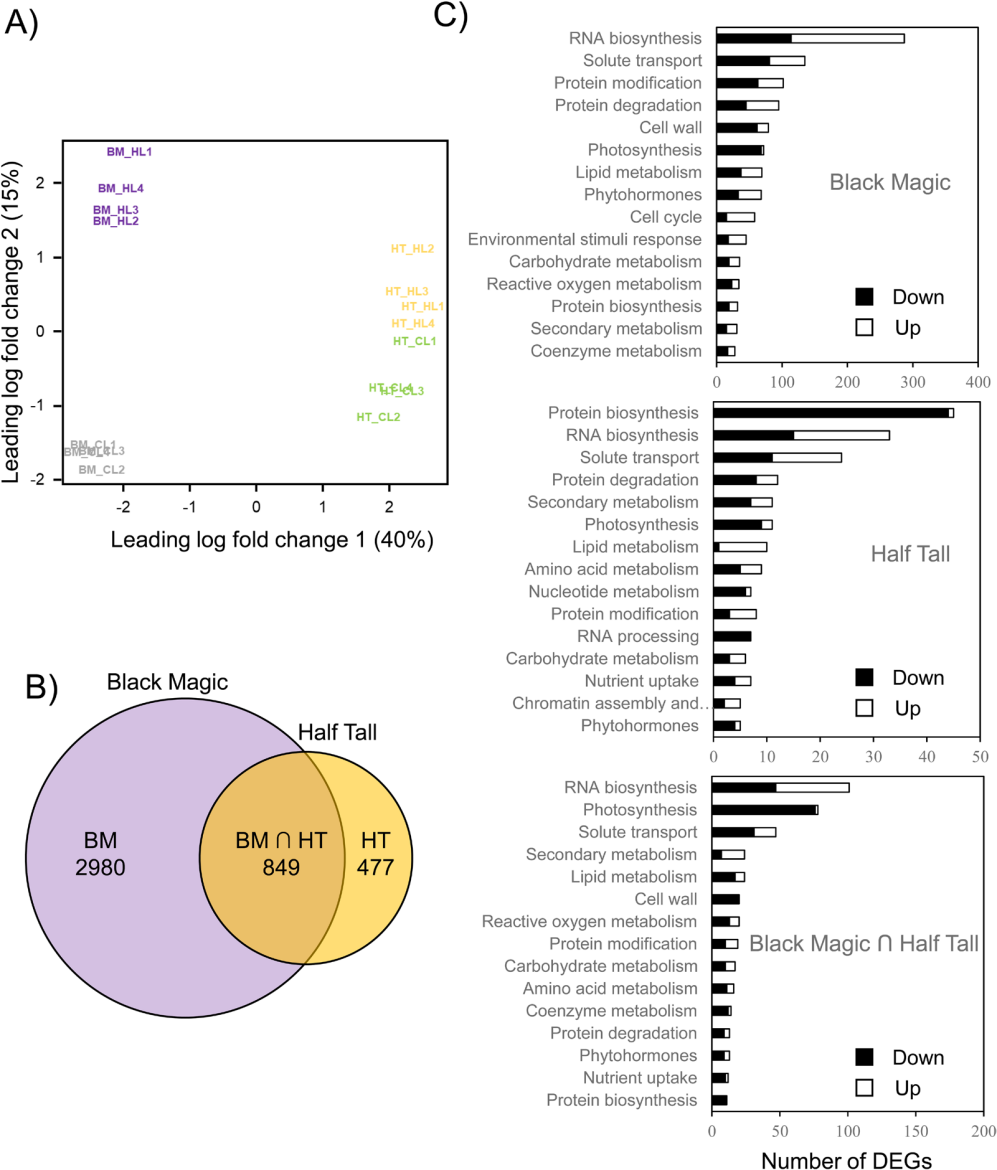
RNA-seq analysis of kale cultivars Half Tall and Black Magic. The RNA-seq reads generated from the Illumina platform were mapped against cabbage reference genome (Cai *et al.*, 2020) to identify differentially expressed genes (DEGs) under high light conditions (HL; 800 µmol photons m^−2^ s^−1^ at 26 °C) compared with control light (CL; 130 µmol photons m^−2^ s^−1^ at 22 °C) conditions [DEGs: false discovery rate (FDR)<0.05, log_2_(fold-change)>1] in Black Magic (BM) and Half Tall (HT). (A) Multidimensional scaling plot of the top 500 genes displaying the highest fold changes. Each treatment group, defined by cultivar and treatment, consists of four biological replicates. (B) Venn diagram depicting unique and shared sets of DEGs under HL between BM and HT. (C) MapMan bin enrichment in each section of the Venn diagram. The source data are presented in Supplementary Dataset S5.

To identify candidate genes and pathways involved in the differential accumulation of anthocyanin and GSLs in the two kale cultivars, RNA-seq analysis was performed with the same leaves that were used in the metabolite analysis. The sequence reads were mapped against 29980 CDS sequences from *Brassica oleracea* var. *capitata* (cabbage) from Cai *et al.* (2020) to identify genes differentially expressed in high light and elevated temperature (Supplementary Table S1: log_2_(fc)>1, FDR<0.05). A MDS analysis was used to visualize the similarity of the individual replicates between and within the cultivars and under different lighting conditions. Altogether 500 DEGs, which showed the highest expression levels among the four experimental sets, were used in the analysis. The MDS plot showed that the light conditions and the cultivars were clearly separated by a leading log_2_(fc) dimension 1 and 2, which explained 55% of the total variance (Fig. 6A).

Analysis of the RNA-seq data revealed genotype-specific responses to growth under high light and elevated temperature conditions. Statistical analysis of differential gene expression in plants grown under high light and elevated temperature as compared with control conditions identified 3829 DEGs in Black Magic and 1326 DEGs in Half Tall. These included 849 genes that were differentially expressed under high light and elevated temperature in both cultivars (Fig. 6B). MapMan bin enrichment analysis of the DEGs revealed that genes related to RNA biosynthesis, photosynthesis, and solute transport were highly enriched in both cultivars (Fig. 6C). About 96% of the DEGs in the photosynthesis bin were down-regulated in both cultivars, while over 60% of the DEGs involved in secondary metabolism were up-regulated in high light conditions.

Examination of possible high-light-associated temperature effects in the kale transcriptomes showed that homologues of the Arabidopsis gene *HEAT SHOCK PROTEIN* (*HSP*) *101* (*HSP101*) and *HEAT SHOCK PROTEIN 22* (*HSP22*) were up-regulated in both Black Magic and Half Tall grown under high light conditions (Supplementary Fig. S4). A homologue of the Arabidopsis gene *HEAT SHOCK FACTOR A1A* (*HSFA1A*) was up-regulated only in high-light-grown Black Magic. The increased expression of *HSFA1A* was accompanied by decreased expression of genes encoding homologues for HSP70 and HSP90, which act as negative regulators for *HSFA1* and *HSFA2* in Arabidopsis (Ohama *et al*., 2017). Homologues for the Arabidopsis *HEAT-SHOCK TRANSCRIPTION FACTOR A2* (*HSFA2*) or the heat responsive *DEHYDRATION-RESPONSIVE ELEMENT BINDING PROTEIN 2A* (*DREB2A*) were not found among significantly expressed genes in either of the kale cultivars (Supplementary Fig. S4). Taken together, the high light conditions, with a recorded 4 °C temperature increase, provoked some temperature effects in gene expression, which were not manifested as clear responses to heat.

### Up-regulation of anthocyanin biosynthesis-associated genes under high light conditions

**Fig. 7.**
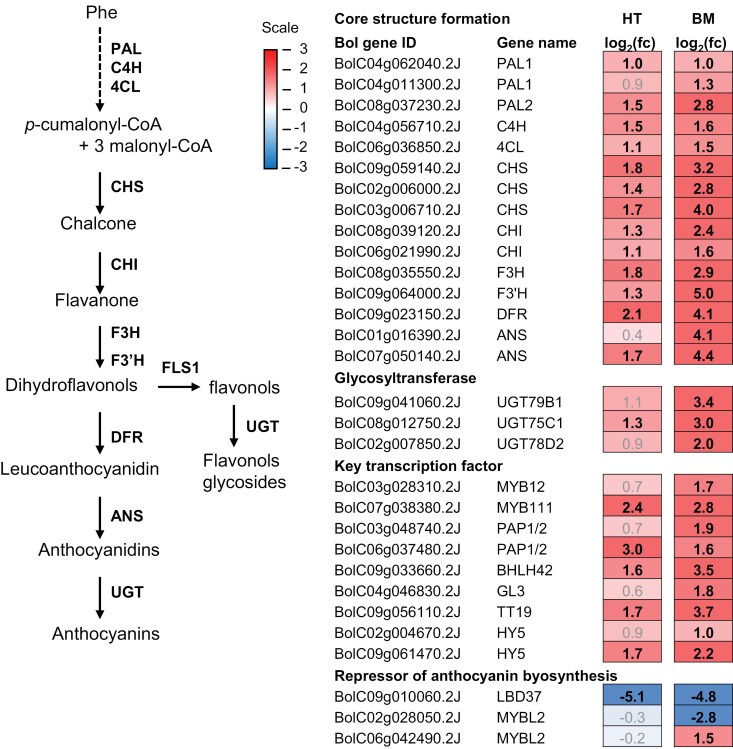
Expression of genes involved in phenylpropanoid biosynthesis in Half Tall (HT) and Black Magic (BM) kale cultivars. Simplified phenylpropanoid biosynthesis pathway with selected genes, based on sequence similarity to Arabidopsis reference genes. The dotted lines indicate multiple reactions. Log_2_(fc) represents logarithmic fold-change in gene expression. Genes significantly and differentially expressed under high light conditions (HL; 800 µmol photons m^−2^ s^−1^ at 26 °C) are indicated in black and bold (log_2_(fc)>1, false discovery rate (FDR)<0.05). The source data are presented in Supplementary Dataset S5. ANS, anthocyanin synthase; BHLH, bHLH transcription factor; C4H, cinnamate 4-hydroxylase; CHI, chalcone isomerase; CHS, chalcone synthase; DFR, dihydroflavonol-4-reductase; F3H, flavanone 3-hydroxylase; F3ʹH, flavonoid 3ʹ-hydroxylase; GL3, glabra 3; HY5, elongated hypocotyl 5; LBD37, lateral organ boundary domain 37; MYB, MYB transcription factor; PAL, phenylalanine ammonia lyase; PAP, production of anthocyanin pigment 1/2; TT19, transparent testa 19; UGT, UDP-glucosyltransferase.

Black Magic accumulated high contents of anthocyanins, especially under high light conditions (Fig. 3). Therefore, we asked whether genes involved in anthocyanidin biosynthesis were differentially expressed between the kale cultivars, using the annotation data available from the Brassicaceae Database (BRAD: https://brassicadb.org/). We were able to map RNA-seq reads to 36 *Brassica oleracea* gene sequences, which showed sequence homology to Arabidopsis genes involved in the phenylpropanoid pathway. In both cultivars, genes encoding the key enzymes and transcription factors of the core phenylpropanoid pathway were significantly up-regulated under high light and elevated temperature, as compared with control light conditions (log_2_(fc)>1, FDR<0.05; Fig. 7). In contrast, *LATERAL ORGAN BOUNDARY DOMAIN37* (*LBD37*), encoding a repressor of anthocyanin biosynthesis, was down-regulated (Fig. 7). However, comparison of gene expression levels between the cultivars, using one-way ANOVA, revealed a significantly higher expression of phenylpropanoid pathway-associated genes in Black Magic grown under the high light conditions (*P*=8.01 × 10^−7^). PRODUCTION OF ANTHOCYANIN PIGMENT 1 and 2 (PAP1 and PAP2) are R2–R3 MYB family transcription factors that positively regulate anthocyanin biosynthesis in Arabidopsis (Borevitz *et al.*, 2000). We identified two *AtPAP1/2* homologues that were significantly more highly expressed under high light conditions. The *PAP1/2* homologue *BolC06g037480* displayed a more than 3-fold increase in Black Magic and 8-fold increase in Half Tall under high light conditions. Another *PAP1/2* homologue, *BolC03g048740*, was mapped in both cultivars, but this gene was significantly up-regulated only in Black Magic, with almost 4-fold increase in expression under high light conditions (Fig. 7). The increased expression of anthocyanin-related genes in high light conditions was well in line with the high anthocyanin content of Black Magic but did not explain the low abundance of anthocyanins in leaves of Half Tall (Fig. 3).

### Down-regulation of indole glucosinolate pathway-associated genes under high light conditions

**Fig. 8.**
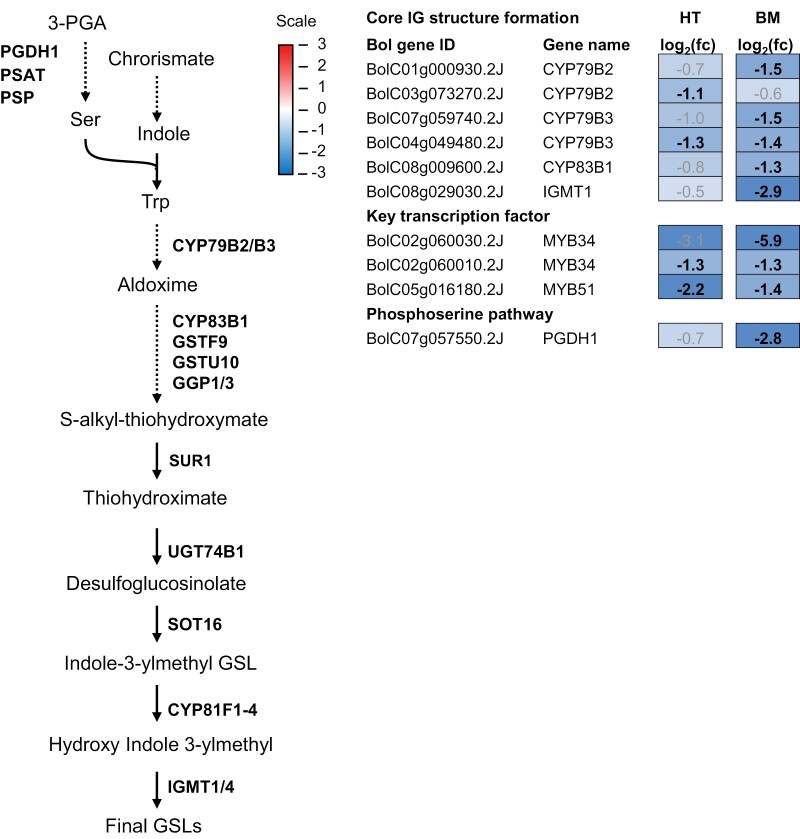
Expression of genes involved in indole glucosinolate biosynthesis in Half Tall (HT) and Black Magic (BM) kale cultivars. Simplified indole glucosinolate (IG) biosynthesis pathway with selected genes, based on sequence similarity to Arabidopsis reference genes. The dotted lines indicate multiple reactions. Log_2_(fc) represents logarithmic fold changes in gene expression. Genes significantly and differentially expressed under high light conditions (HL; 800 µmol photons m^−2^ s^−1^ at 26 °C) are indicated in black and bold (log_2_(fc)>1, false discovery rate (FDR)<0.05). The source data are presented in Supplementary Dataset S5. 3-PGA, 3-phosphoglyeate; CYP, cytochrome P450 family; GSL, glucosinolate; IGMT1/4, indole glucosinolate *O*-methyltransferase 1/4; PGDH1, phosphoglycrate dehydrogenase 1; PSAT, phosphoserine aminotransferase; PSP, phosphoserine phosphatase; SOT16, sulfotransferase 16; SUR1, superroot 1; UGT74B1, UDP-glucosyl transferase 74B1.

The concentrations of Met and Ser were reduced in high light conditions and were significantly correlated with the total concentration of indole GSLs only in Black Magic (Fig. 5). Therefore, we asked whether there were cultivar-specific changes in the expression of genes involved in indole GSL biosynthesis (Fig. 8). The RNA-seq reads mapped to 10 genes that, based on homology with Arabidopsis genes, were annotated as genes involved in indole GSL biosynthesis (log_2_(fc)>1, FDR<0.05). In Half Tall, four indole GSL-related DEGs were identified and found to be down-regulated in high light (Fig. 8). Eight down-regulated indole GSL-related DEGs were identified in Black Magic (Fig. 8). Of note, the *PHOSPHOGLYCERATE DEHYDROGENASE1* (*PGDH1*) homologue *BolC07g057550*, a key enzyme of the phosphoserine pathway, displayed an 8-fold down-regulation in HL-acclimated Black Magic (log_2_(fc)=−2.8, FDR<0.05).

### Alterations in aliphatic glucosinolate related gene expression

**Fig. 9.**
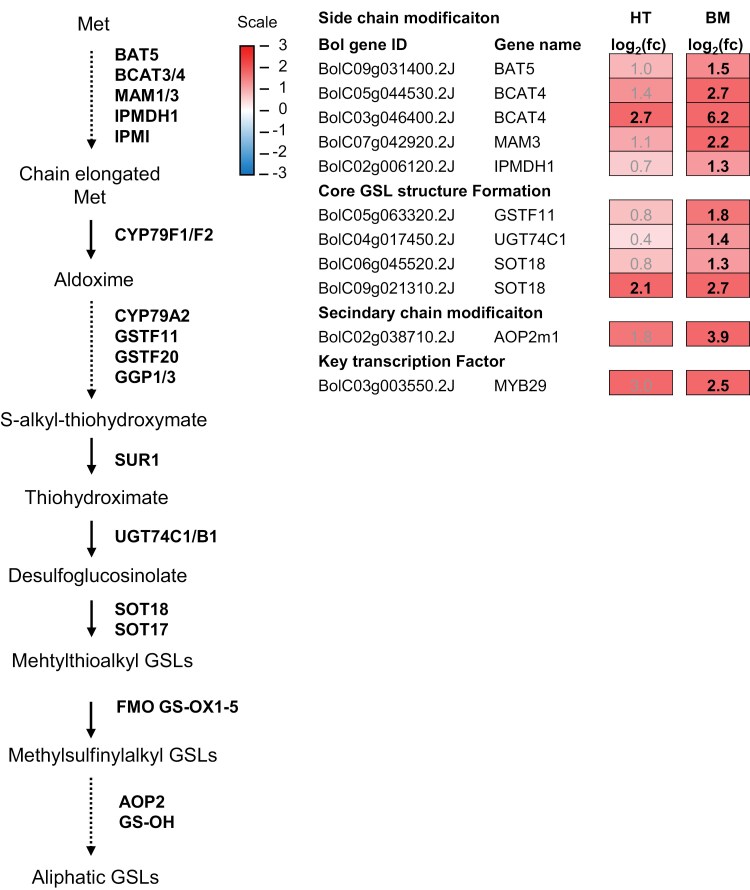
Expression of genes involved in aliphatic glucosinolate (GSL) biosynthesis in Half Tall (HT) and Black Magic (BM) kale cultivars. Simplified aliphatic GSL biosynthesis pathway with selected genes, based on sequence similarity to Arabidopsis reference genes. The dotted lines indicate multiple reactions. Log_2_(fc) represents logarithmic fold changes in gene expression. Genes significantly and differentially expressed under high light conditions (HL; 800 µmol photons m^−2^ s^−1^ at 26 °C) are indicated in black and bold (log_2_(fc)>1, false discovery rate (FDR)<0.05). The source data are presented in Supplementary Dataset S5. AOP2, alkenyl hydroxyalkyl producing 2, BAT5, bile acid transporter 5; BCAT3/4, branched-chain aminotransferase 3/4; CYP79A2, cytochrome P450 79A2; FMO GS-OX1-5, flavin-monooxygenase glucosinolate *S*-oxygenase 1–5; GGP1/3, gamma-glutamyl peptidase 1/3; GS-OH, glucosinolate hydroxylase; GSTF11/20, glutathione-*S*-transferase; IPMDH1, isopropylmalate dehydrogenase 1; IPMI, isopropylmalate isomerase; MAM1/3, methylthioalkymalate synthase-like 1/3; SOT17/18, glucosinolate sulfotransferase 17/18; SUR1, superroot 1.

We identified three aliphatic GSL biosynthesis-associated DEGs (log_2_(fc)>1, FDR<0.05) in Half Tall and 11 DEGs in Black Magic (Fig. 9). All the identified DEGs involved in aliphatic GSL biosynthesis were up-regulated in high light conditions. The side chain modification for production of sinigrin (2PROP GSL), gluconapin (3BUT GSL) and subsequently progoitrin (2*R*-OH-3BUT GSL) is catalysed by a 2-oxoglutarate-dependent dioxygenase encoded by *ALKENYL HYDROXALKYL PRODUCING* 2 (*AOP2*) in Arabidopsis (Hall *et al.*, 2001; Kliebenstein *et al*., 2001c; Li and Quiros, 2003). As these GSL structures were detected only in Half Tall, the composition of aliphatic GSLs in the cultivars differed with respect to enzymatic steps carried out by *AOP2* (Supplementary Fig. S3). In *Brassica oleracea* var *capitata* (cabbage), three Arabidopsis *AOP* homologues, *BolAOP2*, *BolAOP2m1*, and *BolAOP2m2* were identified by Liu *et al.* (2014). *BolAOP2m1* and *BolAOP2m2* were described as non-functional *AOP2* due to the presence of premature stop codons, while no *AOP3* homologues were identified in the cabbage (Liu *et al.*, 2014). Based on the sequence information reported in Liu *et al*. (2014), we identified three kale *AOP* homologues, including *BolC02g038710* (*AOP2*), *BolC03g033940* (*AOP2m1*), and *BolC09g00235* (*AOP2m2*). Only the putative *BolC03g033940* (*AOP2m1*) was significantly higher expressed in Black Magic grown under high light conditions (Fig. 9).

The composition of aliphatic GSLs in the kale cultivars also differed with respect to number of carbon side chains, which is determined by differential actions of METHYLTHIOALKYLMALATE SYNTHASE-LIKE (MAM) isoforms (Supplementary Fig. S2) (Benderoth *et al.*, 2009). A putative *MAM3* homologue *BolC07g042920.2J* was significantly up-regulated in Black Magic under high light conditions (Fig. 9). *MAM1/2* homologues were not found among significantly expressed genes in either of the kale cultivars.

## Discussion

Photosynthetic organisms undergo coordinated adjustments in gene expression and metabolism to optimize their fitness in the prevailing growth environment (Aro *et al.*, 1993; Muller *et al*., 2001; Miyake, 2010; Kono *et al.*, 2014; Tiwari *et al.*, 2016; Gu *et al.*, 2017). Under natural conditions, changes in light intensity are commonly seen as environmental stress, while in indoor cultivation, alterations in supplemental lighting may be applied to improve the nutritional quality of crops. However, the basic understanding of how growth conditions affect the chemical composition of different crops is still limited. Here we report genotype-dependent metabolic responses that influence the composition of GSLs and anthocyanins under contrasting lighting conditions in two kale cultivars commonly consumed in Europe.

### Acclimation to high light and elevated temperature involves genotype-dependent regulation of pigmentation in kale cultivars

The photoprotective metabolites of plants have health-promoting nutritional effects in humans and the growth light environment can therefore directly impact the nutritional value of leafy vegetables (Verkerk *et al.*, 2009; Dinkova-Kostova and Kostov, 2012). The beneficial pigments include carotenoids and anthocyanins with antioxidant properties, and particularly Black Magic kale was rich in these compounds when grown under high light and elevated temperature (Fig. 3). The pale green cultivar Half Tall, in contrast, did not trigger the accumulation of these pigments even under high light conditions, despite doubling of the daily light integral (Fig. 3). In line with the high anthocyanin content, a larger number of genes involved in the phenylpropanoid pathway were significantly up-regulated in Black Magic, compared with Half Tall (Fig. 7). We identified two *PAP1/2-like* genes, encoding homologues for the MYB family of transcription factors that positively regulate anthocyanin biosynthesis in Arabidopsis (Borevitz *et al.*, 2000). *BolC06g037480.2J* was significantly expressed in both kale cultivars, while *BolC03g048740.2J* was only expressed in Black Magic (Fig. 7). Hence, *BolC03g048740.2J* may be the main functional *PAP1/2* behind the high anthocyanin levels of Black Magic (Fig. 3). The observed differences in anthocyanin-related gene expression were not accompanied by increased pigmentation of the Half Tall cultivar (Figs 3, 7). Flavonol derivatives could form another sink for metabolic precursors of the phenylpropanoid pathway in high light conditions (Fig. 7; Stracke *et al*., 2007). This assumption is supported by up-regulation of *MYB111*, a positive regulator of flavonol biosynthesis, in both cultivars (Fig. 7).

In Arabidopsis, anthocyanin biosynthesis is controlled by a complex regulatory cascade that responds to both temperature and light. Growth at 28 °C represses anthocyanin biosynthesis through a mechanism involving CONSTITUTIVE PHOTOMORPHOGENIC 1 (COP1)-mediated degradation of ELONGATED HYPOCOTYL 5 (HY5) (Kim *et al.*, 2017; LaFountain and Yuan, 2021). HY5 is a transcription factor that promotes anthocyanin biosynthesis by activating *miR858a*, which in turn targets the anthocyanin biosynthesis repressor protein MYBL2 (LaFountain and Yuan, 2021). In our experiments on kales, the combination of high light and elevated temperature did not result in down-regulation of *HY5*. In contrast, homologues for *HY5* were up-regulated in both kale cultivars when grown under high light conditions at 26 °C, as compared with control light conditions at 22 °C (Fig. 7). Kale homologues for genes encoding the AtMYBL2 repressor protein, *BolC06042490* and *BolC02g028050*, were not among the DEGs in Half Tall. In Black Magic, both genes were differentially expressed, *BolC06042490* being up-regulated and *BolC02g028050* down-regulated, in high light conditions, compared with the control condition (Fig. 7). Within a putative regulatory cascade, the highly expressed HY5 homologues may function as activators, while the low-expressed BolC02g028050 may represent the AtMYBL2-like repressor of anthocyanin biosynthesis in the purple-coloured Black Magic leaves (Figs 2, 7).

### Serine biosynthesis may limit the production of indole glucosinolates in Black Magic

Biosynthesis of specialized metabolites is tightly connected with primary metabolism, since they share metabolic precursors for biosynthetic pathways (Wink, 2010). The biosynthesis of GSLs relies on the availability of Ser, Met, and Trp, the latter of which derives from the shikimate pathway that also provides metabolic precursors for the biosynthesis of phenylpropanoids (Hirai *et al.*, 2007; Zimmermann *et al.*, 2021). In both Black Magic and Half Tall kales, indole GSLs formed the major constituent of the GSL profiles (Fig. 4). In control light conditions, the content of indole GSL in Black Magic was two times higher than that of Half Tall (Fig. 4), suggesting that Black Magic may require higher amounts of amino acid pre-cursors for the biosynthetic processes. Moreover, in Black Magic, the content of indole GSLs was significantly reduced under high light conditions (*P*=0.014), contrasting with anthocyanin content, which increased in high light conditions (*P*=0.015, Fig. 4). These metabolic changes were reflected by down-regulation of indole GSL-related genes and up-regulation of anthocyanidin-related genes under high light conditions (Figs 7, 8). It is therefore possible that Black Magic limited the biosynthesis of indole GSLs to enhance the biosynthesis of anthocyanins under high light conditions. A similar conclusion, that indole GSL biosynthesis limits phenylpropanoid accumulation, was drawn for Arabidopsis (Kim *et al*., 2015). Half Tall, in contrast, did not show light condition-dependent changes in the levels of these metabolites (Figs 3, 4).

In plants, l-Ser is predominantly synthesized by the phosphoserine pathway (Benstein *et al*., 2013; Okamura and Yokota-Hirai, 2017; Zimmermann *et al*, 2021), which operates in plastids where it represents a deviation from the plastidial glycolysis (Igamberdiev and Kleczkowski, 2018). The phosphoserine pathway is composed of three enzymatic reactions. First, phosphoglycerate dehydrogenase 1 (PGDH1) converts 3-phosphoglycerate to 3-phosphohydroxypyruvate, which is subsequently converted by phosphoserine aminotransferase to phosphoserine and 2-oxoglutarate. Using Glu as an amino-group donor, phosphoserine is converted to Ser by a phosphoserine phosphatase (PSP). Arabidopsis mutants deficient in *PGDH1* and *PSP* were embryo-lethal, indicating that the phosphoserine pathway is essential for plant viability (Benstein *et al*., 2013). *PGDH1*-silenced Arabidopsis plants showed reduced amounts of Trp and indole GSL under high CO_2_ conditions, where photorespiratory generation of Ser was inactive (Benstein *et al*., 2013; Zimmermann *et al.*, 2021).

We found that the declines in indole GLSs and Ser in Black Magic correlated with down-regulation of *PGDH1* of the phosphoserine pathway in high light conditions (Tables 2, 3; Fig. 8). Expression of a *PGDH1* homologue (*BolC0705550*) was significantly decreased in Black Magic (log_2_(fc)=−2.8, *P*<0.01), but not in Half Tall under high light conditions (Fig. 8). Such cultivar-specific decline in *PGDH1* transcript abundance could lower the activity of the phosphoserine pathway in Black Magic, resulting in lower Ser and indole GSL contents (Table 3; Fig. 4; Supplementary Dataset S3). Down-regulation of indole GSL biosynthesis, in turn, would allow redirecting C and N resources for anthocyanin biosynthesis though to the phenylpropanoid pathway. Deepening the understanding of cultivar-specific metabolic interactions could offer a means for controlling Ser biosynthesis and thereby manipulating indole GSL and anthocyanin contents in *Brassica* crops.

### Lighting conditions and selection of genotype offer means for cultivation of healthier *Brassica* crops

The biosynthetic machinery of plants is highly responsive to the growth environment, and the metabolite profiles can therefore be non-invasively manipulated by changes in light and temperature conditions (Cargnel *et al.*, 2014). In this study, GSL profiling provided insights to the nutritional qualities of kale cultivars. Growth of the purple cultivar Black Magic under high light conditions promoted the accumulation of nutritionally beneficial aliphatic GSLs and anthocyanins, while the unfavourable GSL structures remained below the limits of detection (Figs 3, 4; Table 2).

The Met-derived aliphatic GSLs form an important group of natural compounds in the family *Brassicaceae* (Windsor *et al*., 2005). In Black Magic, the major GSL species consisted of the health-promoting glucoraphanin (4MSB GSL) and gluconasturtiin (2PE GSL), and their contents further increased when the plants were cultivated under high light conditions (Supplementary Fig. S3; Table 2). Both Half Tall and Black Magic displayed increase in the transcript abundance for a *MYB29* transcription factor in high light conditions (Fig. 8). This is notable, since *MYB29* serves as activator of short-chained aliphatic GSL biosynthesis in Arabidopsis (Sønderby *et al.*, 2007; Beekwilder *et al*., 2008). Accordingly, growth under high light conditions resulted in an increase in total aliphatic GSLs in both cultivars (Table 2). In Black Magic, the increase in aliphatic GSLs was accompanied by up-regulation of *BCAT4-like* transcripts (Fig. 9), suggesting that increased allocation of Met to GSL biosynthesis could support elevated steady-state levels under high light conditions.

Enzymatic activation of glucoraphanin and gluconasturtiin to their respective isothiocyanates yields metabolites with health-beneficial properties stemming from their anticarcinogenic and chemoprotectant activities (Cheung and Kong, 2010; Jiang *et al.*, 2018). Currently, their potential effects on different cancer types are a matter of extensive study (Castro *et al.*, 2019; Mitsiogianni *et al.*, 2019; Upadhyaya *et al.*, 2019; Yin *et al.*, 2019; Orouji *et al.*, 2023). Sulforaphane, the isothiocyanate derived from glucoraphanin, has also been proposed as a potential therapy for precluding vascular complications in diabetes (Yamagishi and Matsui, 2016).

Among the GSL structures with harmful effects, progoitrin has been associated with a bitter taste, while long feeding periods may cause goiter in animals (Greer, 1957; Felker *et al.*, 2016). Remarkably, in Black Magic the levels of progoitrin were below detection in both light conditions studied (Supplementary Fig. S3). In contrast, Half Tall accumulated progoitrin in similar levels under both light conditions (Table 2; Supplementary Fig. S3).

A highly relevant polymorphic locus controlling GSL profiles is *GSL-AOP* (Magrath *et al.*, 1994; Mithen *et al.*, 1995). Its protein operates downstream of the biosynthesis of the GSL core structure, and its presence or absence determines whether a given species or cultivar predominantly accumulates hydroxyalkyl GSLs, alkenyl GSLs or methylsulfinyl GSLs (Kliebenstein *et al.*, 2001a, b, 2007). Therefore, the *AOP2* locus essentially determines the biosynthetic capacity for progoitrin. In Half Tall, the presence of 2PROP GSL (sinigrin; alkenyl GSL) pointed to the presence of a functional AOP2 enzyme in this cultivar (Table 2; Supplementary Fig. S3), albeit our RNA-seq analysis failed to map mRNA for *AOP2* isoforms (Fig. 9). In contrast, Black Magic accumulated methylsulfinyl GSL in the form of 4MSB GSL (glucoraphanin; Table 2; Supplementary Fig. S3), which was not further converted to other GSL structures, possibly due to the absence of an enzymatically functional AOP2 enzyme in this cultivar.

In conclusion, we observed complex metabolic responses that rely on the interplay between biosynthetic pathways in kale leaves. Characterization of curly leaved cv. Half Tall and a lacinato type cv. Black Magic kales revealed genotype-dependent differences in specialized metabolites, notably anthocyanins and GSLs, which are highly relevant to human nutrition. Improving the understanding of the metabolic interconnections that affect the structural diversity of GSLs may pave the way for traditional breeding or biotechnological engineering of GSL contents and their pungent catabolites in *Brassica* crops (Petersen *et al.*, 2018; Kumar *et al.*, 2019). In addition to the genetic and biochemical foundations of GSL metabolism (Kumar *et al.*, 2019), optimized light and temperature conditions can be applied to modulate the GSL profiles to increase the contents of beneficial aliphatic GSL compounds, while decreasing those with deleterious effects. In all, the lighting conditions can significantly impact the accumulation of beneficial metabolites in commercially valuable cultivars of *Brassica* species.

## Supplementary data

The following supplementary data are available at *JXB* online.

Fig. S1. LC-MS analysis of Black Magic anthocyanins.

Fig. S2. Profiles of indole glucosinolates in Black Magic and Half Tall kales.

Fig. S3. Profiles of aliphatic glucosinolates in Black Magic and Half Tall kales.

Fig. S4. Expression level of genes involved in light and heat stress in Black Magic and Half Tall kales acclimated to high light conditions.

Table S1. Number of differentially expressed genes in differentially light-acclimated kales.

Dataset S1. Spectrophotometric quantification of total carotenoid and anthocyanin contents in Half Tall and Black Magic kale cultivars.

Supplementary Dataset S2. Anthocyanin derivatives identified in kale extracts using UPLC-MS/MS.

Dataset S3. Amino acids and glucosinolates identified in kale extracts using UPLC-MS/MS.

Dataset S4. Correlation analysis of amino acid and glucosinolate data.

Dataset S5. Functional annotation of kale differentially expressed genes.

erae169_suppl_Supplementary_Figures_S1-S4

erae169_suppl_Supplementary_Table_S1_Datasets_S1-S5

## Data Availability

The RNA-seq data was deposited in Gene Expression Omnibus with the BioProject ID PRJNA1013003 (https://www.ncbi.nlm.nih.gov/sra/PRJNA1013003).

## Abbreviations:

2PE: gluconasturtiin, 2-phenylethylglucosinolate GSL
2PROP: sinigrin, 2-propenyl GSL
2*R*-2OH-3BUT: progoitrin
2(*R*)-2-hydroxy-3-butenyl: GSL
3BUT: gluconapin, 3-butenyl GSL
3MSP: glucoiberin, 3-methylsulfinylpropyl GSL
3MTP: glucoiberverin, 3-methylthiopropyl GSL
4MO-I3M: 4-methoxyglucobrassicin, 4-methoxyindol-3-ylmethyl GSL
4MSB: glucoraphanin, 4-methylsulfinylbutyl GSL
4MTB: glucoerucin, 4-methylthiolbutyl GSL
5MSP: glucoalyssin, 5-methylsulfinylpentyl GSL
AOP2: alkenyl hydroxalkyl producing 2
CL: control light growth condition
DEG: differentially expressed gene
I3M: glucobrassicin, indol-3-ylmethyl GSL
GSL: glucosinolate
HL: high light growth condition
MAM: methylthioalkylmalate synthase-like
MDS: multidimensional scaling
NMO-I3M: *N*-methoxyindol-3-ylmethyl GSL, neoglucobrassicin.
