# Supplementary material for: Growth conditions trigger genotype-specific metabolic responses that affect the nutritional quality of kale cultivars
Source: J Exp Bot. 2024 Apr 17;76(5):1427–45. doi: 10.1093/jxb/erae169 (PMC11906305; doi:10.1093/jxb/erae169)
Supplement: erae169_suppl_Supplementary_Figures_S1-S4 [file erae169_suppl_supplementary_figures_s1-s4.pdf]

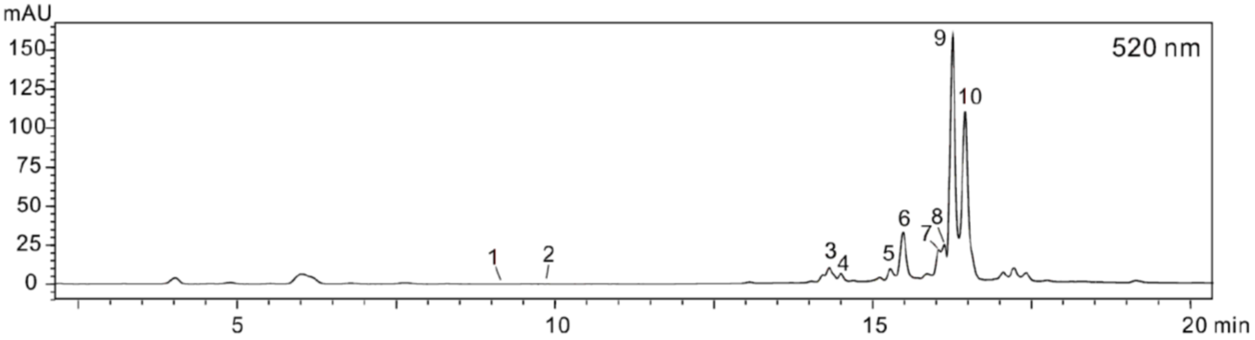

Supplemental Figure S1. Representative chromatogram of the LC-MS analysis of cv. Black Magic kale anthocyanins. Orders refer to retention time, ordinates refer to absorption intensity. Peaks position are marked with number 1 to 10, and their tentative identities are listed in Table 1.

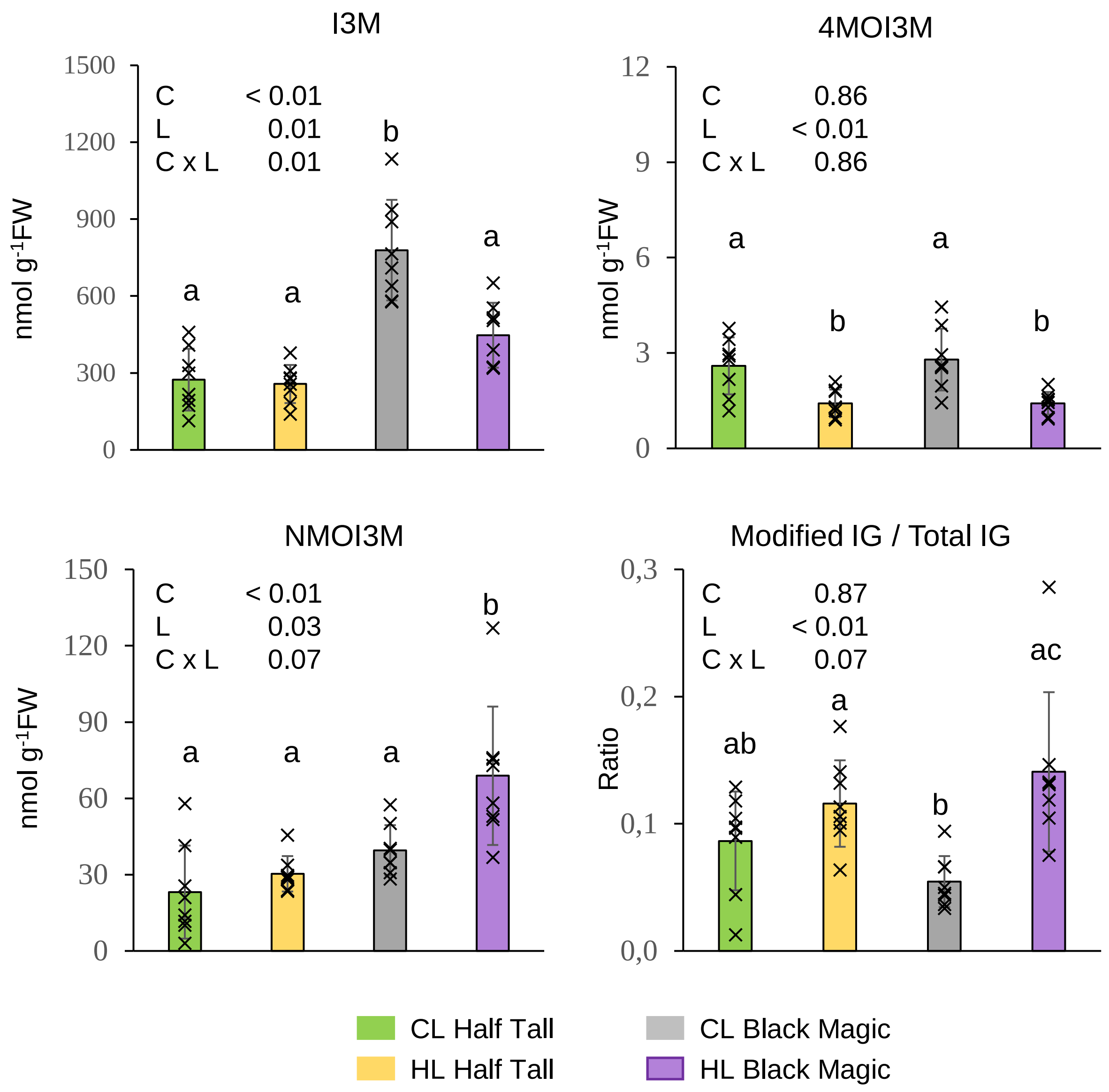

Supplemental Figure S2. Profiles of indole glucosinolates.
Kale cultivars Half Tall and Black Magic were grown under 130 µmol photons $m^{-2}s^{-1}$ at 22°C (CL) or 800 µmol photons $m^{-2}s^{-1}$ at 26°C (HL). Data is shown as mean +/- standard deviation (n=8). The p values from the Two-Way ANOVA (~cultivar (C) + light condition (L) + C x L) are indicated in the upper left corner. For post-hoc pairwise comparisons, P values were corrected for multiple comparisons using Bonferroni adjustment. Different letters indicate statistically significant differences (significance threshold, p=0.05). I3M, indol-3-ylmethylglucosinolate; 4MOI3M, 4-methoxy-indol-3-ylmethylglucosinolate; 1MOI3M, 1-methoxy-indol-3-ylmethylglucosinolate; modified IG, sum of 4MOI3M and 1MOI3M; total IG, total indolic glucosinolates.

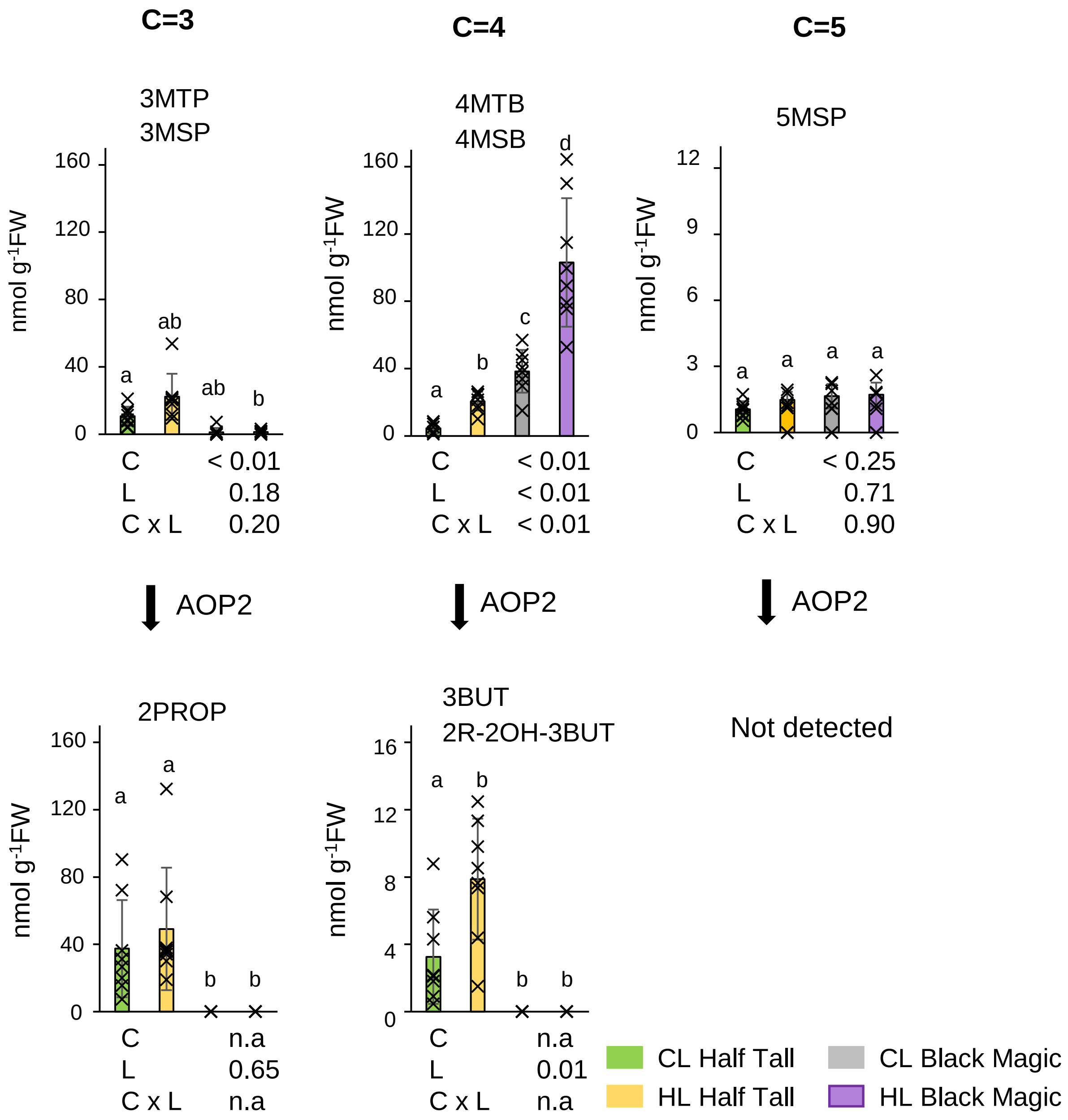

Supplemental Figure S3. Profiles of Aliphatic glucosinolates.

Kale cultivars Half Tall and Black Magic were grown under 130 µmol photons $m^{-2}s^{-1}$ at 22°C (CL) or 800 µmol photons $m^{-2}s^{-1}$ at 26°C (HL). Data is shown as mean +/- standard deviation (n=8). The p values from the Two-Way ANOVA (~cultivar (C) + light condition (L) + C x L) are indicated in the upper left corner. For post-hoc pairwise comparisons, P values were corrected for multiple comparisons using Bonferroni adjustment. Different letters indicate statistically significant differences (significance threshold, p=0.05). Aliphatic glucosinolate derivatives with different side chains found in kale extracts and a schematic representation of structural modifications by 2-oxoglutarate-dependent dioxygenase (AOP2). 3- carbon side chain (C = 3); 3MTP (3-methylthiopropyl GSL), 3MSP (3-methylsulphinylpropyl GSL; glucoiberin) and 2PROP GSL (2-propenyl GSL; sinigrin), 4-carbon side chain (C = 4): 4MTB (4-methylthiolbutyl GSL; glucoerucin), 4MSB (4-methylsulphinylbutyl GSL; glucoraphanin), 3BUT (3-butenyl GSL; gluconapin), 2R-2OH-3BUT (2(R)-2-hydroxy-3-butenyl GSL; progoitrin). 5-carbon side chain (C = 5): 5MSP (5-methylsulphinylpentyl GSL; glucoalyssin).

Light Stress

Bol gene ID	Gene name	HT log$_2$(fc)	BM log$_2$(fc)
BolC03g000050.2J	LEA1	0.4	-0.1
BolC08g010580.2J	OHP2	-0.1	0.2
BolC06g011700.2J	OHP2	-0.2	0.1
BolC04g068370.2J	GOLS1	**1.8**	-0.3
BolC04g001200.2J	SOLS1	**1.7**	2.3
BolC05g057370.2J	APX1	0.3	0.6
BolC05g005690.2J	APX1	0.0	**0.5**
BolC01g052680.2J	APX2	0.7	3.0
BolC07g056430.2J	APX6	0.3	0.5
BolC07g057940.2J	APX3	-0.2	-0.3
BolC01g003060.2J	APX5	0.1	-0.6
BolC09g034700.2J	sAPX	**-0.7**	-0.6
BolC06g048670.2J	tAPX	-0.8	**-1.6**
BolC02g018170.2J	tAPX	0.2	-0.5

Heat Stress

Bol gene ID	Gene name	HT log$_2$(fc)	BM log$_2$(fc)
BolC01g011120.2J	HSFA1A	0.3	**0.7**
BolC03g029720.2J	HSFA2	0.2	0.7
BolC06g031680.2J	HSP101	**0.5**	**1.2**
BolC06g045760.2J	HSP101	**0.9**	**3.6**
BolC03g031730.2J	HSP22	**3.2**	**3.6**
BolC01g024560.2J	HSP70	0.0	**-1.7**
BolC07g046440.2J	HSP70	-0.5	**-1.3**
BolC01g018470.2J	HSP90	0.0	**-0.5**
BolC08g017190.2J	HSP90	-0.9	-1.3
BolC07g051220.2J	HSP90	-0.1	-0.1
BolC02g002320.2J	DREB2	-0.5	-0.6
BolC09g066540.2J	DREB2	0.4	0.8

Supplemental Figure S4. Expression level of genes involved in light and heat stress.

Kale cultivars Half Tall and Black Magic were grown under 130 µmol photons $m^{-2}s^{-1}$ at 22°C (CL) or 800 µmol photons $m^{-2}s^{-1}$ at 26°C (HL). Log(fc) represents logarithmic fold changes of gene expression. Genes significantly differentially expressed in high light condition are indicated with black and bold (log_2(fc)>1, FDR <0.05). The source data is presented in Supplemental data set S5. APX1, Ascorbate Peroxidase 1; DREB2, Dehydration-Responsive Element-Binding Protein 2**;** GOLS1, Galactinol Synthase 1; HSP, Heat Shock Protein; HSFA, Heat Shock Transcription Factor A ; LEA, Late Embryogenesis Abundant 1; OHP2, One-Helix Protein 2; sAPx, Stromal Ascorbate Peroxidase; tAPx, Thylakoid-Bound Ascorbate Peroxidase,
